# Individual variation in life-history timing: synchronous presence, asynchronous events and phenological compensation in a wild mammal

**DOI:** 10.1098/rspb.2023.2335

**Published:** 2024-04-17

**Authors:** Roxanne S. Beltran, Raquel R. Lozano, Patricia A. Morris, Patrick W. Robinson, Rachel R. Holser, Theresa R. Keates, Arina B. Favilla, A. Marm Kilpatrick, Daniel P. Costa

**Affiliations:** ^1^ Department of Ecology and Evolutionary Biology, University of California Santa Cruz, Santa Cruz, CA 95060, USA; ^2^ Institute of Marine Sciences, University of California Santa Cruz, Santa Cruz, CA 95060, USA; ^3^ Department of Ocean Sciences, University of California Santa Cruz, Santa Cruz, CA 95060, USA

**Keywords:** individual, variation, life history, timings, synchronous, presence

## Abstract

Many animals and plants have species-typical annual cycles, but individuals vary in their timing of life-history events. Individual variation in fur replacement (moult) timing is poorly understood in mammals due to the challenge of repeated observations and longitudinal sampling. We examined factors that influence variation in moult duration and timing among elephant seals (*Mirounga angustirostris*). We quantified the onset and progression of fur loss in 1178 individuals. We found that an exceptionally rapid visible moult (7 days, the shortest of any mammals or birds), and a wide range of moult start dates (spanning 6–10× the event duration) facilitated high asynchrony across individuals (only 20% of individuals in the population moulting at the same time). Some of the variation was due to reproductive state, as reproductively mature females that skipped a breeding season moulted a week earlier than reproductive females. Moreover, individual variation in timing and duration within age-sex categories far outweighed (76–80%) variation among age-sex categories. Individuals arriving at the end of the moult season spent 50% less time on the beach, which allowed them to catch up in their annual cycles and reduce population-level variance during breeding. These findings underscore the importance of individual variation in annual cycles.

## 1. Introduction

**Figure 1.**
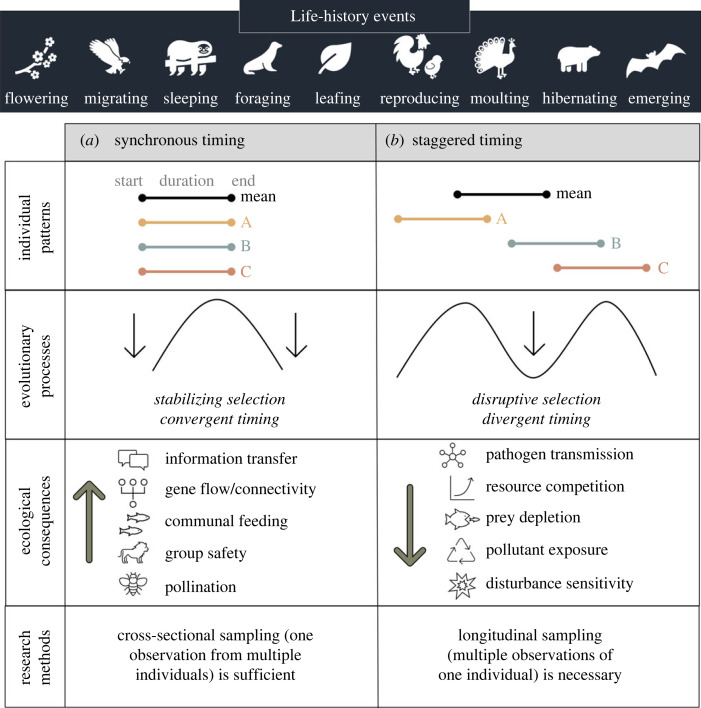
Evolutionary drivers and ecological implications of daily and seasonal activity timing across individuals. Synchronous timing across individuals (*a*), which may result from stabilizing selection, facilitates many beneficial ecological consequences. Staggered timing across individuals (*b*), which may result from disruptive selection, can also benefit individuals by reducing negative ecological consequences. The degree of synchronicity determines whether longitudinal sampling is required for measuring the duration of a daily or seasonal activity, or whether cross-sectional sampling is sufficient. In nature, individual activities lie on a continuum between synchronous and staggered timing, and ecological consequences may vary in direction and magnitude depending on context and scale (e.g. large prey groups that form during breeding or foraging could both attract and swamp predators).

A major question in biology is how rapid changes in environmental conditions such as temperature and productivity [1] shift life-history timing in wildlife [2]. The survival and reproductive success of individuals depend on their ability to align life-history events, including breeding, foraging and migrating, with the availability of resources such as food and mates. In addition, variation in life-history timing among individuals can have myriad impacts [3]. For example, synchronous life-history events in a population, such as migration, can increase both within-species behavioural connections, including social networks, and between-species interactions, such as pollination or transmission of infectious diseases [4]. Synchronicity can also alter the intensity of ecosystem processes, including the addition of nutrients and contaminants through migration and death, defaecation and moulting (figure 1*a*) [5–7]. In contrast, asynchronous life-history timing, such as staggered arrival of individuals to a feeding area, can reduce competition for prey and attraction of predators (figure 1*b*). However, individual-level timing data are difficult to collect. Studies of life-history timing that focus on population averages ignore individual variation and obscure the ecological implications of variation in synchronicity. Moreover, snapshots of timing from many individuals pieced together may vastly overestimate life-history event duration if individuals are asynchronous (figure 1). A critical step in understanding the response of species, communities and ecosystems to a changing world is to examine how life-history timing varies across ages, sexes and individuals.

Moulting is a common annual process in birds and mammals that impacts other life-history events through behavioural changes [8], higher energy requirements [9] and increased physiological stress [10]. The moulting process also influences survival and reproductive success by facilitating thermoregulation, camouflage [11], mate acquisition [12], contaminant offloading [13] and wound healing through changes in colour, texture and thickness of fur and feathers [14]. Temporal variation of life-history events, including moult, is often studied with cross-sectional sampling designs [15,16] in unmarked individuals [17] through methods including remote cameras [18]. Though these methods limit disturbance [19] and facilitate valuable population-level inferences [20], they cannot be used to quantify variation in life-history timing across individuals, sexes and ages of many species [21,22], which inhibits an understanding of moult in individuals and synchrony among individuals. For example, cross-sectional studies often show that life-history events occur over a long duration, which begs the question of whether individual-level life-history events are long or whether many individuals with staggered timing are contributing to long population-level durations. Additionally, few studies have determined which factors lead to individual variation in moult timing, especially in mammals. Some critical unanswered questions include: how synchronous are life-history events? Is asynchrony a result of weak selective pressure, or does selective pressure vary across demographic groups with different energetic and social requirements? Thus, determining the timing and duration of the moult has strong implications for understanding natural processes and their sensitivities across scales, from individuals to populations to ecosystems.

Here, we quantify intraspecific variation in moult timing of northern elephant seals, *Mirounga angustirostris*. Elephant seals are ideal candidates for studying moult progression because the inefficiency of perfusing the skin for fur replacement while immersed in the ocean constrains them to moult on land during extended periods ('haul-outs') [23,24]. Their dense aggregations during seasonal haul-outs along with the conspicuous shedding pattern in which the thick epidermal layer attached to fur roots peels off [21,22] (‘catastrophic moult’ [14]) allow for large numbers of individual animals and their moult progression to be observed longitudinally [25]. In addition to facilitating fur replacement, the moult haul-out allows seals to rest and recover from the physiologically intense foraging trip and avoid predation by sharks and orcas [26] (although note that both predator species appear to aggregate near seal haul-out areas [27]). However, there is a cost to time spent onshore, as it reduces body condition by precluding foraging [28]. During the haul-out, slower moult progression is also thought to increase energy expenditure and lead to substantial heat loss [14], which could have carry-over effects to other components of the annual cycle. This poses an evolutionary tug-of-war on the moult and produces a potential mechanism by which individuals with varying body condition and pregnancy states may differ in the amount of time they spend onshore for the moult and the rate at which they replace their fur.

We used repeated observations of uniquely tagged individual seals to estimate the start date and duration of the moult and moult haul-out periods, along with variation among individuals. Specifically, we ask: (1) how long does the moult last, and how much do individuals differ in moult duration? (2) Is timing of the moult less synchronous than reproductive timing? (3) Do age and reproduction status of females affect when they moult? We hypothesized that moult duration would be relatively short compared with the moult haul-out duration and that moult timing would vary less across individuals than breeding timing in this highly polygynous species. We also hypothesized that reproductive state and age would affect moult timing, as discovered in previous studies [9,29,30]. Together, these objectives will help uncover the ecological implications of individual variation in life-history phenology.

## 2. Methods

### (a) Study site and system

Northern elephant seals come ashore (hereafter, ‘haul-out’) twice per year, once for breeding during the winter and once for moulting in the spring/summer [28]. Adult females perform a foraging trip after the breeding season lasting 2–3 months, and a much longer foraging trip after the moult season lasting 7 months and travelling up to 10 000 km from colonies on the west coast of North America to remote pelagic feeding sites across the Pacific Ocean [31,32]. The capital breeding strategy of elephant seals allows them to acquire needed resources in advance and store them as fat reserves for extended haul-outs on land, during which they do not consume prey [33,34]. Much research has been devoted to the timing of pupping and mating; females arrive in mid-January, give birth 5 days later, then nurse their pups for 27 days [35]. Much less attention has been devoted to the timing of the moult; we know the broad seasonality, with females and juveniles of both sexes moulting in the spring and males moulting in the summer, and a population average of moult haul-out duration for adult females [36,37]. Critical knowledge gaps remain, including how moult strategies vary across individuals and whether the moult duration accounts for the time individuals spend hauled out onshore.

We studied adult female and juvenile northern elephant seals as part of the long-term research programme at Año Nuevo Reserve, California, USA (37.12°N, 122.31°W) [31]. We placed unique alpha-numeric plastic cattle ear tags (mostly Dalton Jumbo Roto tags) in the interdigital webbing of the hind flippers of young-of-the-year seals soon after birth [38]. Tags persist for years, allowing us to identify individuals throughout seasons and over their lifetimes [38], by using cameras and binoculars from a distance (approx. 3+ metres) or approaching seals cautiously to read worn and faded tags. The colony population includes approximately 10 000 seals of which approximately 10–20% have flipper tags. The maximum number of seals on the beach at any given time is approximately 3500 individuals. Field crews searched the colony on most days between December and June each year (2016–2022) to record repeated observations of each flipper-tagged seal. We had observations on 75% of all days (47–69 days each year) during the core 81-day window (09 Apr to 28 Jun) when animals were observed in the process of moulting. The probability of detecting animals and recording moult progression is high due to the extensive effort and conspicuous nature of the moulting process; however, seals often form huddling aggregations during the moult haul-out [39] such that part of the flipper tag or body (and therefore moult progression) are sometimes obscured.

### (b) Observing moult progress

**Figure 2.**
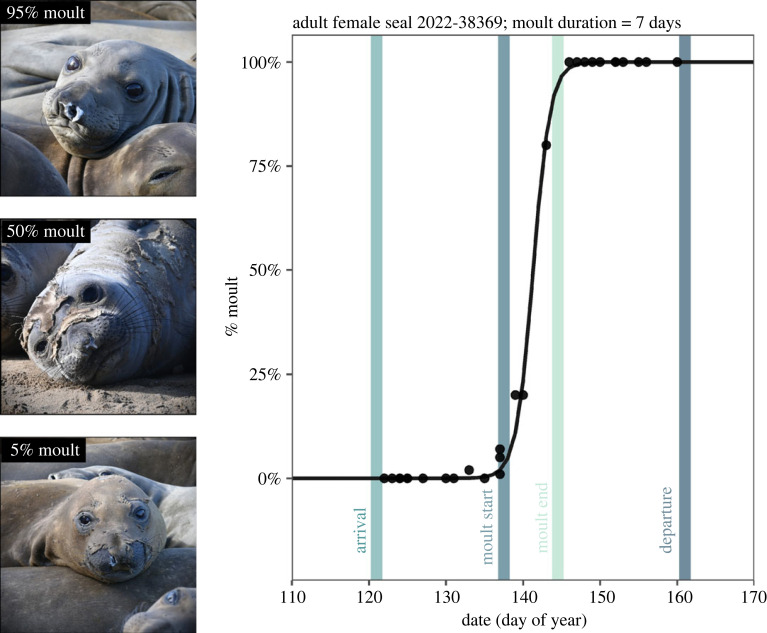
Timing of moult and haul-out for one representative adult female elephant seal #38369 in the year 2022. The black line shows a Bayesian likelihood logistic function fitted to *N* = 29 observations of the proportion of the body that had moulted (% moult, black dots). The coloured vertical lines denote the estimates of each event of interest for this individual. Arrival and departure dates are estimated by correcting the first and last observations, respectively, for detection probability. Seal images show moult progression (photographs by Dan Costa under (National Marine Fisheries Service (NMFS) permit 23188).

The seals’ faces (especially eyes and whiskers) and hind flippers are the first to moult. Often animals lose fur more quickly on the ventral side of their body, likely due to warmth and friction while moving across sandy surfaces, however, the pattern of fur shedding can be patchy and varies between individuals. Therefore, the percentage of the body that has moulted is a better indicator of moult progression in this species than qualitative moult scores that have been used in other species [9]. For every sighting of a tagged seal, the per cent of old fur that had peeled away to reveal the new coat was estimated to the closest integer, 0–100 (figure 2). Old fur varies from light brown to yellow across individuals and peels in sheets to reveal the new fur below, which is shiny grey (figure 2). Patches of old fur and new fur are thus readily identifiable, allowing for a quantitative estimate of the per cent of new fur visible. Over 7 years, we made 13 969 moult observations of 1178 annual moult cycles (one animal in 1 year) of 643 unique animals. Each year included an average of 168 moult cycles (ranging from 77 in 2017 to 275 in 2021). Moult cycles for many animals were repeatedly observed across several study years: 1 year = 376 animals, 2 years = 132 animals, 3 years = 69 animals, 4 years = 26 animals, 5 years = 19 animals, 6 years = 15 animals, 7 years = 6 animals.

### (c) Consistency of moult scores

We examined consistency in moult scoring among observers by analysing 1220 cases where two or more observers independently assigned a moult percentage to the same animal on the same day (electronic supplemental material). Observations were highly correlated (*R*^2^ = 0.986), and the largest error was mistaking a fully moulted seal for one that had not moulted, or *vice versa*; in both cases, there was no peeling fur. This error occurred in less than 3% of all cases when the percentage was either 0% or 100%. These duplicate cases were included in the analysis.

### (d) Modelling moult progress

We modelled moult progress by fitting a logistic curve through the observations of fraction moulted (0–1) as a function of time, defining time as the day of each sighting (the exact time of day was not used because most observations were in the early morning) (figure 2).

To determine moult parameters (e.g. moult duration and timing), we ran the model with moult cycles that met three criteria: (1) there were at least six observations during the progression of the moult, (2) there was at least one observation of moult less than 10% and (3) at least one greater than 90%. Models were run separately for reproductive age adult females (age four or older; *N* = 782 year-individual combinations), juvenile females (younger than 4 years old and had not produced any pups; *N* = 232) and juvenile males (younger than 3 years old; *N* = 164). We did not include adult males in our analysis because they moult in a separate season (summer rather than spring) [36] and the small number of individually tagged males that reach adulthood precludes a robust analysis. The sample of adult females that skipped reproduction during the prior breeding season (age four or older females that did not attend the breeding season; *N* = 109) were included in the reproductive age adult female model and their model results were visualized separately *post-hoc*. During the breeding season, we estimated a 60% detection probability for adult females present [38], so the skip adult females (females that didn't breed in a year) probably included some animals that did have a pup during the previous winter and/or attended a different breeding colony that year. Despite this caveat, we refer to the two groups as reproductive females and skip females. Some seals (*N* = 229) were of unknown age but were observed as adults during the year of observation and were thus included in the reproductive age adult female model.

We estimated each individual's moult date, defined as the day on which the fitted moult curve was at 50%, and the duration of the moult, defined as the time it took the fitted logistic curve to pass from 5% to 95% (figure 2). We estimated moult start date for each individual by subtracting half the moult duration from the estimated 50% moult date. The hyper-parameters included the mean moult date across individuals and years, as well as a mean date for each individual and each year, and the same for moult duration. Hyper-parameters also included explicit estimates of variance among individuals and years (see the electronic supplemental material for additional details). A challenge arose in estimating moult timing and duration for individuals with few sightings, especially if observations did not span the full moult and include intermediate moult values. We thus adopted a multi-level modelling approach, fitting data from all animals at once while adding an overarching hyper-distribution across individual logistic parameters. This multi-level approach produced a direct estimate of variation among individuals and between years [40,41]. We examined year-to-year variation in moult timing by examining variation across the 7 years of data. We used Bayesian posteriors to estimate credible intervals of each parameter as well as derived parameters (i.e. those calculated from original parameters).

### (e) Parameter fitting and model verification

We examined model fit to the data for each seal graphically (see the electronic supplemental material). In most cases, the fitted model reliably described moult progression data and arrival-departure dates formed a smooth distribution. We examined outliers and discovered some cases where unusual fits were observed (e.g. unusually short moult durations due to uncertain field observations), but none were removed from the analyses because uncertainty was a part of the observation process. Details of our examination of outliers are presented in the electronic supplemental material.

### (f) Modelling arrival and departure

With rare exceptions, moulting animals remain on the colony throughout the haul-out period (even though their moult duration comprises a small fraction of the moult haul-out period). Therefore, the moult haul-out duration can be estimated from the pre-moult arrival date until the post-moult departure date. However, there may be a delay between true arrival and the first observation depending on the frequency of researcher observations and the animal's detection probability. Therefore, we modelled detection probabilities separately for arrival date and departure date. Animals selected for the haul-out duration model came from a subset of the moult timing model in which 95% credible intervals of moult start date was narrower than 6 days (*N* = 431 adult females, 130 juvenile females, 102 juvenile males). Haul-out duration parameters were fitted by the same Bayesian Monte Carlo method used in the moult model. The key parameters were arrival and departure dates for each individual plus a calculated haul-out duration (departure date minus arrival date, in days). Similar to the moult model, a multi-level approach was used, with individual and year effects incorporated. Model details are presented in the electronic supplemental material.

### (g) Synchrony across the moulting population and annual cycle

We measured synchrony as the maximum fraction of the entire population that was present or moulting on any one day. We estimated the fraction of animals present on the colony by subtracting the cumulative fraction of the population that have departed from the cumulative fraction that have arrived each day [42]. Likewise, we estimated the fraction of animals moulting by subtracting the cumulative fraction of the population that had finished their moult from the cumulative fraction that had started. Because we found no year-to-year variation in moult timing, we used dates of arrival, moult and departure across all years to produce the estimate.

To quantify the degree of variation in life-history timing, we retrieved individual-level data on timing of eight life-history events from various sources: arrival from the long foraging trip [43], birth (adding 6 days to arrival; [37]), weaning and departure for the short foraging trip (adding 27 days to birth; [35]), arrival from the short foraging trip (this manuscript), moult start (this manuscript), moult end (this manuscript) and departure for the long foraging trip (this manuscript). We then obtained standard deviations for the breeding season using published literature [37,43] and all moult life-history events (this manuscript) to compare variance across the annual cycle.

All statistical analyses were run in R version 4.2.1 [44]. We used generalized linear mixed effects models with gamma distributions (log link) from the package ‘lme4’ to compare moult duration, moult timing and haul-out duration across age, sex and reproductive state categories (electronic supplementary material, tables S1–S3), and to quantify the relationship between haul-out timing and haul-out duration (electronic supplementary material, tables S4–S7). To partition variance between age-sex categories, year and individual, we ran a model of each moult metric as a function of the interaction between age-sex categories and year, with individual as a random effect, and then calculated the fixed effects variance explained.

## 3. Results

### (a) Moult timing

**Figure 3.**
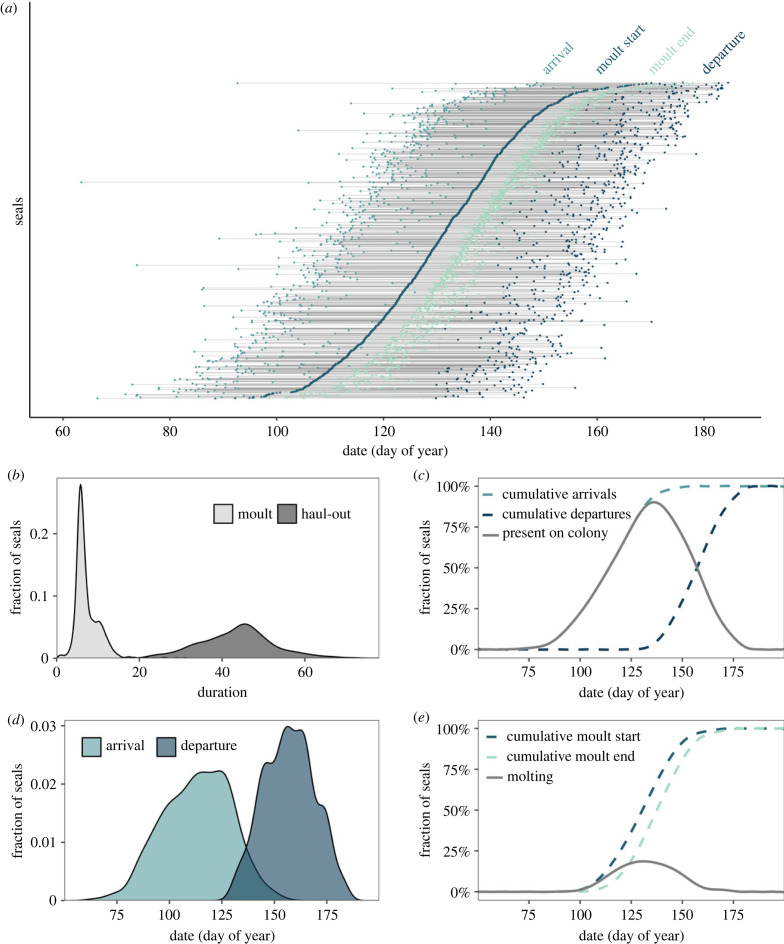
For each of the *N* = 1178 seals across age and sex classes, estimated arrival and departure dates that demarcate the haul-out duration (black lines), along with modelled moult start and end dates that comprise moult duration (grey lines) (*a*). The inset plots show density plots of moult and haul-out durations (*b*), density plot of arrival and departure dates (*d*), cumulative distributions of arrival and departure dates along with the fraction of seals present at the colony on each day (*c*), and cumulative distributions of moult start dates along with the fraction of seals moulting at the colony on each day (*e*).

**Figure 4.**
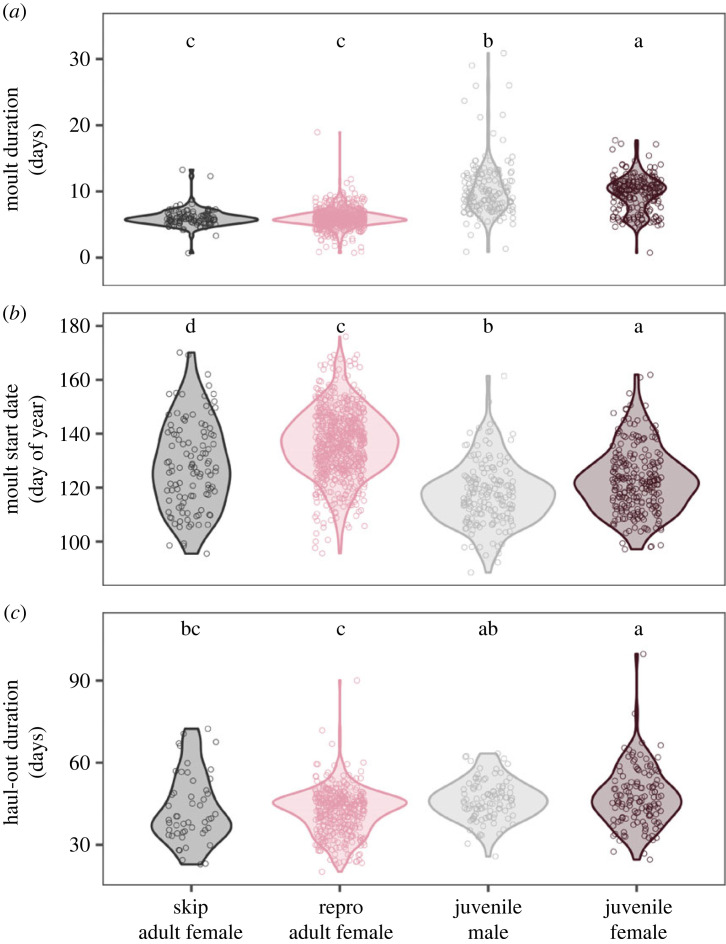
Violin plots and individual values (dots) of moult duration (*a*), moult start date (*b*) and haul-out duration (*c*) for four age-sex categories. Moult is significantly shorter in adult females and later in reproductive adult females as compared with juveniles of both sexes, despite relatively similar haul-out durations. Two or more predictors with the same grouping letter were not different at the *α* = 0.05 level.

There was a wide range of moult start (5% moulted), moult end (95% moulted), haul-out duration and moult duration values across seals (figures 3*a*, 4). The 95% credible intervals of seal arrival dates spanned 58 days from 25 March to 22 May, and the departure dates spanned 45 days from 14 May to 29 June (figure 3*d*). Juveniles arrived first in the spring, with mean arrival dates of 10 April (males) and 13 April (females); they remained hauled out, on average, for 46 days until late May (figure 4*c*). Reproductive adult females hauled out 2 weeks later (mean arrival 01 May), and they remained hauled out for 4 days less (42 days; departing 12 June; figure 4*c*). Reproductive females moulted later as they aged (7 days later across 10 years of age for reproductive females) but skip adult females did not (electronic supplementary material, figure S1). In juveniles, however, the ageing pattern was the opposite; moult start date moved earlier between ages 1 and 2 in both males and females (electronic supplementary material, figure S1). Skip adult females had similar haul-out durations but arrived to moult a week earlier than reproductive adult females. Juvenile males started moulting first (28 April), followed by juvenile females (5 days later), skip adult females (11 days after juvenile males) and adult females (19 days after juvenile males) (figure 4*c*). Skip adult females moulted earlier than reproductive adult females for all ages. Haul-out duration decreased with age in skip adult females but not reproductive adult females (electronic supplementary material, figure S1). The effect of breeding status was especially clear at age three, when non-breeding females moulted 12 days before breeding females (electronic supplementary material, figure S1).

### (b) Moult duration

Averaged across all age and sex categories, moulting started an average of 18 days after arrival (± s.e. 0.20) and animals departed 18 days after moult end (± s.e. 0.22), indicating that moulting is centred in the moult haul-out. Moult progressed quickly after starting (figure 2), and moult duration was significantly shorter in reproductive and skip adult females (mean ± s.e.: 5.95 ± 0.06 days for reproductive females, 6.01 ± 0.16 day for skip females) than juvenile females (9.67 ± 0.17 days) and juvenile males (10.29 ± 0.22 days) (figure 4*a*). Moulting comprised 14% of the adult female haul-out on average compared with 20% and 24% of the juvenile female and juvenile male haul-out, respectively (figure 3*b*).

### (c) Synchrony across the annual cycle

Life-history timing was characterized by a strong lack of synchrony across individuals and age-sex categories; some seals finished moulting and departed for the ocean migration before other seals arrived to moult (figure 3*a*). Individual variation dwarfed age and sex differences in moult-related life-history timing. After accounting for the interaction between year and age-sex category, as well as individual random effects, a large proportion of variation in moult metrics was explained by individual (79.8% in haul-out duration, 76.6% in moult duration and 75.8% in moult start date) (figure 4).

**Figure 5.**
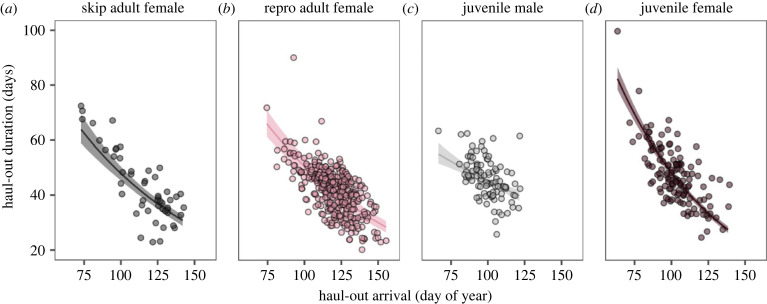
Relationship between haul-out duration and haul-out arrival date for each age-sex category. Across all age-sex categories, seals that arrived for the haul-out later had shorter haul-out durations, presumably to ‘catch up’ in the annual cycle (*p* < 0.05 for all). Lines shown are generalized linear mixed effects models with gamma distributions (log link).

**Figure 6.**
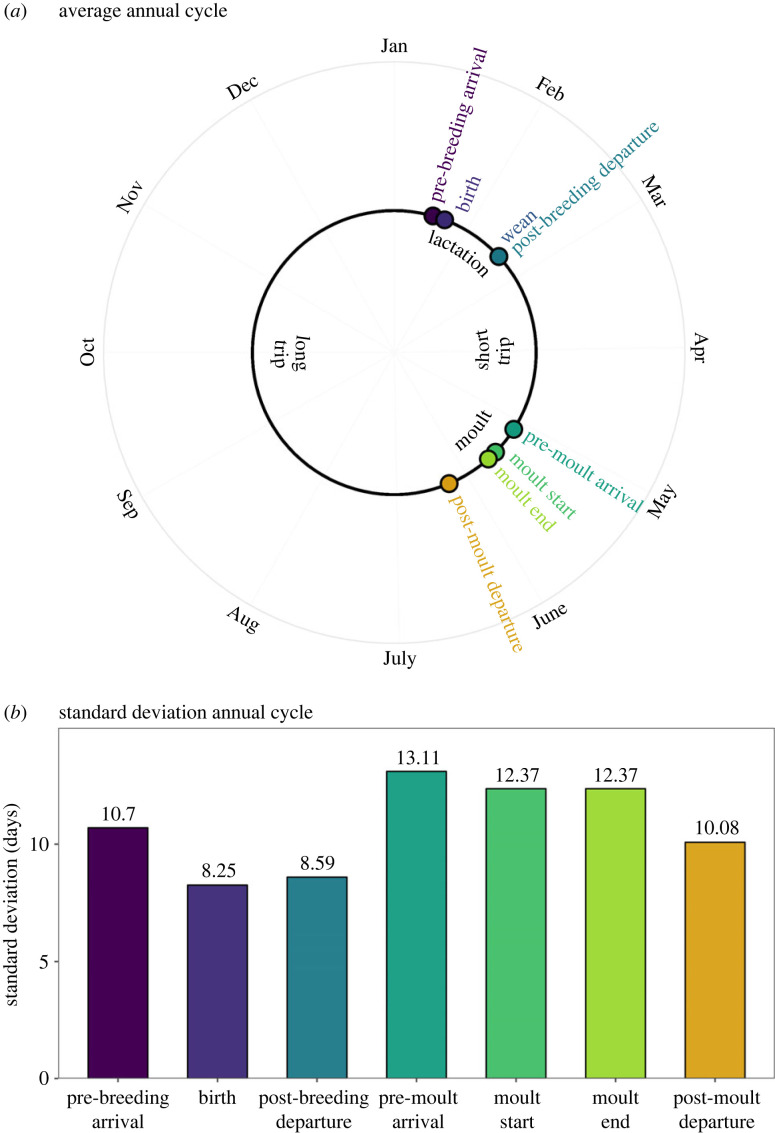
Timing of critical life-history events measured in individual reproductive adult female elephant seals shows a reduction in variance throughout the annual moult and lower variance overall during the breeding season. Average life-history events in panel A are from Condit *et al*. [43] (pre-breeding arrival), Condit *et al*. [37] (pre-birth delay), Costa *et al*. [35] (lactation duration) and this paper (pre-moult arrival, moult start, moult end and post-moult departure). Standard deviation of life-history events in panel B are from Condit *et al*. [43] (breeding arrival), Condit *et al*. [37] (birth), D Costa 2024, personal communication (post-breeding departure) or calculated from this paper (moult parameters).

Across all age-sex categories, seals that arrived later had a shorter haul-out duration (figure 5, electronic supplementary material, tables S4–S7). In adult females, the earliest arrivals stayed on the colony more than twice as long as the latest arrivals (figure 5). This inverse relationship between moult haul-out duration and arrival resulted in diminishing variance from moult arrival to moult start to departure to breeding arrival, which facilitated departure after the moult and synchrony of arrival during the breeding season for reproductive females (figure 6). For adult females, the degree of variation in life-history event timing was markedly lower during the breeding season than the moulting season (figure 6). The standard deviation of dates declined from moult arrival to moult start and end, moult departure and breeding season arrival (figure 6).

### (d) Synchrony across the moulting population

At the peak of the moult haul-out, 90% of seals were on the beach (16 May, figure 3*c*) because the population haul-out period (95% interval 95.9 days) was only twice as long as average individual haul-out durations (43.8 days, figure 3*b,c*). In stark contrast, only 20% of the population were moulting simultaneously at the peak (12 May, figure 3*e*), because the population moult duration (95% interval 61.4 days) was more than eight times longer than the average individual moult duration (7.3 days, figure 3*d,e*). There were no apparent differences in arrival dates, departure dates or synchrony across years (electronic supplementary material, figure S2).

## 4. Discussion

Although variation across individual organisms strongly influences ecology and evolution (figure 1) [45], the magnitude and drivers of individual variation in life-history timing are less well understood than average timing. Elephant seals moult on a regular annual cycle, but we discovered that life-history timing is surprisingly variable and staggered across individuals compared with anecdotal, cross-sectional observations. The moult start and haul-out dates varied widely, even within a single age and sex category; as a result, only 20% of juveniles and adult females moult at the same time (figure 3). Surprisingly, variance across individuals in life-history timing far outweighed variance across age-sex categories, particularly for the haul-out duration (figure 4). Despite extensive variation among individuals in moult timing, 90% of individuals in the population were present at the mid-point of the moult season because the haul-out durations of individual seals are long, and the moult is centred in the haul-out. This is an underestimate of moult timing asynchrony at the population level because adult males (which were not included in this manuscript) moult during the summer, months after the juveniles and adult females. In comparison, during the breeding season, 85–87% of reproductive females are present at the mid-point of the season despite lower variation in arrival date because the breeding haul-out is shorter [37,46].

We found that the fur shedding process accounted for only 16% of haul-out durations and less than 2% of the annual cycle for adult females. Elephant seals appear to push the physiological boundaries of moult progression, with shorter visible moult durations than all previously studied marine mammals (electronic supplementary material, table S5 in [9]) and birds [14,47]. Visible fur replacement, however, does not include the entire moult, because fur and skin growth are initiated prior to visible shedding, and new fur continues to grow after shedding is complete [48]. The timing and duration of the entire fur replacement process can only be determined from skin biopsies, as subdermal follicle growth precedes visible fur loss [48,49]. Thus, our estimates of moult duration only include the visible moult (from onset to termination of fur loss) and are likely to underestimate the thermal and energetic costs of moulting.

We found considerable variability in moult timing resulting from breeding status. Females that did not give birth in the prior breeding season, and all juveniles, moulted earlier. The same pattern has been found in seals [9] and birds [29]. For reproductively successful elephant seals, delaying moult could mean a shorter foraging trip afterwards, during the crucial period when females are diving nearly continuously and consuming thousands of fish daily [31,50] to support gestation and prepare for the upcoming lactation period [51]. The relative synchrony of haul-out timing but asynchrony of moult timing could mean that predator aggregation potential is still high, as other studies have found [e.g. 27]. Moreover, the input of mercury from moulted skin into coastal waters (8× higher during moult compared with seal-free sites [52]) could be prolonged and the energetic costs of temperature anomalies during the moulting process could differently impact individuals with varying moult progression [53].

Many animals and plants in both temperate and tropical climates have annual life cycles organized around annual reproduction. Other phases of the life cycle must conform to the annual rhythm, even those not tied to reproduction, such as migration, leaf fall or moult (figure 1). A widely held hypothesis is that the annual cycle is arranged so that offspring are produced when conditions are most favourable for their survival [54,55], and this suggests that other phases of the cycle are not subject to strong selection for timing, only to fit in somewhere else. We discovered that for breeding females, the standard deviation of moult arrival is higher than all other moult events (moult start and moult departure) as well as the arrival of the breeding season (figure 6*b*), which suggests that the moult haul-out may be the most flexible portion of the elephant seal annual cycle [21]. Interestingly, the short, pre-moult foraging trip adds substantially to intraspecific variation in life-history timing [56], whereas the moult haul-out reduces intraspecific variation. The latter is due to the negative correlation between haul-out arrival date and haul-out duration (figure 5), which results from late arriving animals finishing the moult haul-out quicker to catch up in the annual cycle relative to animals arriving earlier. This also appears to occur during the breeding haul-out [37], although to a much lesser extent. The long foraging trip also reduces variance in population-level timing because elephant seals adjust their movement direction and speed based on their distance from the breeding colony [32], similar to other migrating species [57]. A better understanding of the annual cycle, including the relationship between moult timing and future reproductive success, will elucidate how temporal constraints such as annual cycles that require more than 365 days to complete may force the adoption of non-optimal annual routines, including intermittent breeding [29,58].

We discovered that wide population-level life-history timing due to extreme variation across individuals results in temporal overlap between life-history events. Some seals arrive to start moulting after other seals have already finished moulting and departed for their long foraging migrations. Therefore, in this study system, assumptions made from cross-sectional phenology studies would lead to erroneous inflations of individual-level life-history durations and problematic assumptions of population-level synchrony. In other species, conclusions drawn from cross-sectional studies of unmarked individuals may similarly reflect a population-wide window rather than individual life-history event durations. For example, in mountain goats *Oreamnos americanus*, fur shedding occurs over a 3 month period [59], but individuals may moult for a much shorter period of time within that population-level phenology window. Therefore, researchers should take care to clearly distinguish phenological durations across individuals or populations depending on study constraints.

Future studies could explore the role that genotypes, hormones and reproductive cycles play in driving and maintaining individual differences in vertebrate life-history timing. Our findings underscore that individual variation in life-history timing should be integrated into mechanistic frameworks such as the Population Consequences of Disturbance models, which rely on physiological and behavioural parameterization to understand the demographic implications of anthropogenic disturbances [60]. If links between these state variables and life-history timing are made, targeted management efforts could account for variability in life-history timing across individuals that are more sensitive or hold more reproductive potential. Likewise, models and management efforts should consider the phenotypic flexibility of species and individuals with various states. For example, our work demonstrated some degree of temporal plasticity in elephant seals as later arriving individuals of all age and sex classes accelerated the moult haul-out (figure 5), spending only half the time on the colony relative to early arriving individuals. Morrison *et al*. [61] reported similar flexibility in life-history timing, but by comparing species rather than individuals within species. There are several potential mechanisms driving the flexibility of individual timing, including the role of body condition. For example, individuals that extend their foraging trips and thus arrive later to moult may have reduced body condition [56], and need to depart earlier when their endogenous lipid stores reach low levels. Alternatively, individuals that arrive later may have acquired more resources during the preceding foraging trip, and therefore may be able to moult more efficiently or recuperate more quickly. Both hypotheses could be tested by measuring both body condition and life-history timing of individual seals.

Together, these findings produce valuable insights into intraspecific variation in the timing and duration of life-history events, which play an underappreciated role in community- and ecosystem-level processes (figure 1). Unfortunately, we could not compare the degree of synchrony of elephant seal life-history events to those of other species due to the limited availability of individual-level longitudinal life-history timing studies. Our work emphasizes the importance of adding life-history timing to the critical drivers of ecological processes alongside intraspecific trait variation [62] and individual heterogeneity [63].

An outstanding question is how individual variation in life-history timing could modulate population impacts of seasonal resource pulses or unexpected environmental anomalies (e.g. marine heatwaves) [64]. These events are particularly noteworthy for large marine vertebrates, including sea turtles, seabirds, pinnipeds and polar bears, that leave the marine environment to undertake critical life-history events, including nesting, mating, hibernating and lactating on land. Their movement from marine to terrestrial systems facilitates the flow of nutrients, energy, organic matter, toxins and organisms, which can negatively or positively impact ecosystems depending on the context [65]. Shifting the research paradigm away from averages and toward quantifying variation across individuals will help us understand how synchrony within and across populations may buffer against the impacts of climate change.

## Data Availability

Data and code are available at Dryad: https://doi.org/10.7291/D1C10C [66]. Supplementary material is available online [67].
